# The temporal course of third molar mineralization in a black South African population

**DOI:** 10.1007/s00414-025-03664-2

**Published:** 2025-11-24

**Authors:** Julian Wirtz, Chané Smit, Liam Robinson, Herman Bernitz, Maximilian Timme, Sven Schmidt, Andreas Schmeling

**Affiliations:** 1 Institute of Legal Medicine, University and University Hospital Münster, Röntgenstraße 23, 48149 Münster, Germany; 2 Department of Oral and Maxillofacial Pathology, Faculty of Health Sciences, University of Pretoria, Gauteng, South Africa

**Keywords:** Age estimation, Dental age, Wisdom tooth, OPG, Demirjian stages

## Abstract

There are only a few studies available on the temporal course of third molar mineralization in sub-Saharan Africans. In the present study, 904 orthopantomograms (OPGs) of self-classified black South Africans aged 12–26 years with confirmed dates of birth were examined. 748 OPGs showed at least one evaluable mandibular third molar. The stages of mineralization for teeth 38 and 48 were determined by consensus according to the stage classification by Demirjian et al. (Hum Biol 45:221–227, 1). For stages D to H, the minimum age, maximum age, mean age with standard deviation, and median age with lower and upper quartiles are presented separately for each sex. The mean ages for both sexes for both teeth are above 18 years of age from stage G onwards. The minimum ages in stage H are 17.9 years (tooth 38) and 18.0 years (tooth 48) for males. For females, these values are 18.1 and 17.2 years, respectively. Since the minimum ages are above the values reported in other studies, they cannot be recommended for age assessment practice. In order to avoid stage misclassifications, which are particularly problematic when applying the minimum age principle, experienced examiners should make stage classifications in future studies consensually by.

## Introduction

Authorities and courts commission forensic age assessments to ensure that migrants with unclear age information are neither disadvantaged due to their age nor that procedures dependent on age are carried out incorrectly [1]. In most countries, the forensically relevant age limits are between 7 and 21 years of age [2]. The most important question in age assessment practice concerns the completion of 18 years of age.

If there is a legal basis for X-ray examinations without medical indication, the international and interdisciplinary Study Group on Forensic Age Diagnostics (AGFAD) recommends a three-step procedure for age assessments in adolescents and young adults. The first stage involves a physical examination with an anamnesis. If there are no indications of development-accelerating diseases or medication use, the second stage involves an X-ray examination of the hand and a dental examination with an orthopantomogram (OPG). If the development of the hand skeleton is complete, an additional CT scan of the clavicles is performed in the third stage [3–5].

The main criteria for dental age determination are the eruption and mineralization of the third molars [6–9]. The stage classification according to Olze et al. [10] is recommended for the evaluation of third molar eruption [11]. For the assessment of third molar mineralization, the stage classification according to Demirjian et al. [1] has proven to be the most suitable [12].

Reference studies for forensic age assesment must meet specific requirements [4]. These include an even age distribution of the sample. Since X-ray examinations of healthy individuals for the sole purpose of research are not possible for legal and ethical reasons, and X-ray images taken for medical reasons are therefore used, most of the available studies do not meet the requirement of an even age distribution. Various research groups have shown that an uneven age distribution leads to distortions of the statistical measures (so-called age mimicry) [13, 14]. Therefore, reference studies that meet the requirement of an even age distribution are desirable.

For legal and ethical reasons, overestimating age must be avoided at all costs [15]. Several studies have reported that third molar mineralization occurs earlier in sub-Saharan Africans than in other ethnic groups [16, 17]. Applying reference studies conducted on non-sub-Saharan Africans to sub-Saharan Africans would therefore carry the risk of overestimating age. In numerous African countries, there is no reliable registration of births. Of the 150 million unregistered children worldwide, more than half currently live in sub-Saharan Africa (https://data.unicef.org/resources/the-right-start-in-life-2024-update/). However, reliable birth dates of the subjects are a prerequisite for reference studies on forensic age diagnosis. The aim of the present study is to improve the data basis on the temporal course of third molar mineralization in sub-Saharan Africans with reliable birth dates.

## Materials and methods

For the study, 904 OPGs of self-classified black South Africans aged 12–26 with verified dates of birth were available. The X-rays were taken for medical reasons at the University of Pretoria Oral Health Centre, Pretoria, Gauteng, South Africa, between 2011 and 2022. Table 1 shows the age distribution of the sample, broken down by sex.

**Table 1:** Age and sex distribution of the study population

Age (years)	Males (*n*)	Females (*n*)	Total (*n*)
12	30	28	58
13	30	28	58
14	27	29	56
15	27	26	53
16	27	30	57
17	29	24	53
18	28	33	61
19	29	30	59
20	29	34	63
21	26	32	58
22	29	30	59
23	30	32	62
24	32	30	62
25	41	34	75
26	40	30	70
Total	454	450	904

The inclusion criteria were as follows: (1) no condition that could affect the presence and development of third molars, (2) at least one existing lower third molar. The exclusion criteria were (1) insufficient image quality affecting third molar visualization, (2) tilting of the third molar leading to impaired stage determination, (3) single-rooted third molar, (4) fused tooth roots.

The mineralization status of the lower third molars was assessed using the stage classification by Demirjian et al. [1]. All OPGs were evaluated by three examiners. Two examiners had more than 20 years of experience in assessing third molar mineralization using the Demirjian stages. The third examiner was a doctoral student who was calibrated prior to the evaluations. Where the ratings were found to disagree, the examiners reached a consensus through subsequent discussion of the cases.

All statistical analyses were performed using IBM SPSS Statistics (Version 29.0.0.0, IBM Corp., Armonk, NY, USA). For each Demirjian stage (D–H), and separately for both sexes and teeth 38 and 48, the minimum, maximum, mean age with standard deviation, and median age with lower and upper quartiles were calculated. Descriptive statistics were used exclusively, as the aim of the study was to provide reference data rather than to test specific hypotheses. The data were examined for consistency and plausibility prior to statistical evaluation.

## Results

Of the 904 OPGs available, 748 OPGs showed at least one evaluable mandibular third molar. Table 2 presents the age and sex distribution of the evaluable OPGs. Reasons for non-evaluability were insufficient image quality, tilted teeth, single-rooted teeth, and fused tooth roots.

**Table 2:** Age and sex distribution of the study population with evaluable OPGs

Age (years)	Males (*n*)	Females (*n*)	Total (*n*)
12	11	15	26
13	17	17	34
14	23	24	47
15	19	17	36
16	23	24	47
17	26	21	47
18	24	28	52
19	24	29	53
20	28	34	62
21	23	30	53
22	27	29	56
23	29	26	55
24	26	27	53
25	38	27	65
26	39	23	62
Total	377	371	748

Tables 3, 4, 5 and 6 detail the number of cases, minimum, maximum, mean values with standard deviations, as well as medians with lower and upper quartiles for the age of mineralization of teeth 38 and 48 according to Demirjian’s stages D-H for males and females. Due to the small number of cases, no statistical measures are presented for stages A-C.

**Table 3:** Statistical measures for stages D to H for tooth 38 in males

Stage	*N*	Min	Max	Mean	SD	Median	LQ	UQ
D	14	12.01	14.43	13.32	0.73	13.11	12.84	14.10
E	44	12.34	17.36	14.89	1.22	14.99	14.11	15.81
F	53	13.91	20.54	17.32	1.53	17.30	16.31	18.27
G	65	17.07	25.49	20.37	2.08	20.28	18.64	21.85
H	159	17.86	26.93	23.86	2.21	24.33	22.28	25.81

Ages in years. *SD* standard deviation, *LQ* lower quartile, *UQ* upper quatile

**Table 4:** Statistical measures for stages D to H for tooth 48 in males

Stage	*N*	Min	Max	Mean	SD	Median	LQ	UQ
D	16	12.06	16.02	13.73	1.05	13.80	13.07	14.29
E	43	12.34	16.90	14.64	1.17	14.62	13.69	15.57
F	49	13.91	21.22	17.11	1.51	17.21	16.23	18.00
G	68	16.81	26.00	20.30	2.17	19.89	18.63	21.67
H	159	18.02	26.93	23.95	2.18	24.39	22.31	25.69

Ages in years. *SD*) standard deviation, *LQ*) lower quartile, *UQ* upper quatile

**Table 5:** Statistical measures for stages D to H for tooth 38 in females

Stage	*N*	Min	Max	Mean	SD	Median	LQ	UQ
D	26	12.00	16.18	13.84	1.16	13.90	12.80	14.71
E	40	12.57	26.38	15.29	2.32	15.08	13.73	16.12
F	56	13.48	22.03	17.91	2.07	17.92	16.65	19.31
G	81	16.21	25.17	20.51	2.22	20.38	18.83	21.98
H	128	18.09	26.76	23.39	2.26	23.74	21.95	25.44

Ages in years. *SD* standard deviation. *LQ* lower quartile. *UQ* upper quatile

**Table 6:** Statistical measures for stages D to H for tooth 48 in females

Stage	*N*	Min	Max	Mean	SD	Median	Lower	Upper
D	22	12.00	17.37	13.69	1.36	13.63	12.68	14.61
E	41	12.57	26.38	15.09	2.30	14.81	13.51	15.89
F	57	13.48	21.28	17.43	1.86	17.59	16.23	18.73
G	82	16.21	26.18	20.56	2.05	20.54	19.11	21.93
H	129	17.22	26.88	23.48	2.29	23.99	22.10	25.47

Ages in years. *SD*) standard deviation, *LQ* lower quartile, *UQ* upper quatile

In both males and females, the data reveal that the minimum and maximum values, as well as the mean and median values of chronological age, increase with progressive stages of mineralization. The mean ages for both sexes for both teeth are above 18 years of age from stage G onwards. For males, the minimum age for stage H is 17.9 years for tooth 38 and 18.0 years for teeth 48. For females, the minimum age is 18.1 years for tooth 38 and 17.2 years for tooth 48.

## Discussion

In this study, the mineralization stages of lower third molar of 748 OPGs were evaluated. Despite the retrospective study design, the requirement for an even age distribution in the sample was largely met. Tables 3, 4, 5 and 6 show the minima, maxima, means with standard deviations, and medians with lower and upper quartiles for mineralization stages D to H. When applying these statistical parameters in age assessment practice, it should be noted that the means and standard deviations as well as the medians with lower and upper quartiles for stage H depend on the upper age limit of the sample examined and are therefore not suitable for age assessments.

To the authors’ knowledge, there are only a few studies on the temporal course of third molar mineralization in sub-Saharan Africans. In certain studies involving the relevant population, other methods are used for example, making a direct comparison with the present data impossible [18]. However, there are also some studies that are comparable in terms of population, age range, and methodology. Olze et al. [19] examined third molar mineralization in 474 male and 121 female black South Africans aged 10–26 years using the stage classification of Demirjian et al. [1]. They published mean values with standard deviations and medians with upper and lower quartiles for mineralization stages D-H for both sexes. In a later publication, Olze et al. [10] also reported minimum ages for stages F-H for males in this sample. Cavrić et al. [20] examined the OPGs of 807 males and 953 females aged 6 to 23 years from Botswana. They determined the Demirjian stages A-H for teeth 28 and 38. For stages A-G, sex-separated mean values with standard deviations, medians with upper and lower quartiles, and minima and maxima were reported. For stage H, only the minima were reported. Uys et al. [17] examined the mineralization of teeth 28 and 38 in 287 male and 276 female black South Africans aged 15–23 years. The stage classification used was that of Solari and Abramovitch [21]. This is a modification of the Demirjian stages with the additional stages F1 and G1. For stages D to H, mean values with standard deviations, medians with upper and lower quartiles, and minimum and maximum values were reported for both sexes. Table 7 shows the mean values with standard deviations and the age minima of the present study and the other studies on sub-Saharan Africans for mineralization stages D to H of tooth 38. No mean values with standard deviations are given for stages F and G of the study by Uys et al. [17], as these are not comparable with the other studies due to the stage classification used by the authors.

**Table 7:** Statistical measures (mean ± standard deviation, minimum) in years for stages D to H of tooth 38 of the present study and other studies investigating third molar mineralization in sub-Saharan Africans. Values are rounded to one decimal place

	Present study	Olze et al. [19]/12)	Cavrić et al. [18]	Uys et al. [17]
Stage D males	13.3 ± 0.7, 12.0	13.4 ± 1.6, -	12.7 ± 1.4, 8.8	16.1 ± 0.7, 15.0
Stage D females	13.8 ± 1.2, 12.0	13.6 ± 2.5, -	12.4 ± 1.7, 6.1	16.0 ± 0.9, 15.0
Stage E males	14.9 ± 1.2, 12.3	15.4 ± 2.6, -	15.0 ± 1.5, 11.7	15.7 ± 0.7, 15.0
Stage E females	15.3 ± 2.3, 12.6	15.7 ± 1.8, -	14.8 ± 1.7, 11.2	16.4 ± 1.4, 15.0
Stage F males	17.3 ± 1.5, 13.9	18.6 ± 2.5, 12.8	16.6 ± 1.6, 14.4	Min: 15.0
Stage F females	17.9 ± 2.1, 13.5	17.1 ± 2.5, -	16.9 ± 1.6, 14.2	Min: 15.0
Stage G males	20.4 ± 2.1, 17.1	21.1 ± 2.2, 17.6	18.3 ± 1.6, 14.7	Min: 15.3
Stage G females	20.5 ± 2.2, 16.2	19.6 ± 2.2, -	18.4 ± 1.5, 15.3	Min: 15.0
Stage H males	23.9 ± 2.2, 17.9	22.9 ± 2.4, 17.4	Min: 15.7	21.2 ± 1.8, 16.5
Stage H females	23.4 ± 2.3, 18.1	22.5 ± 2.3, -	Min: 15.1	21.6 ± 1.6, 17.4

When comparing the mean values, the different age intervals of the studies must be taken into account. Knell et al. [13, 22] have shown that the age interval of the study population influences the mean age values if the youngest and oldest individuals who can exhibit the respective stages are not included. Truncating the upper age segment leads to a reduction in the mean ages, while truncating the lower age segment leads to an increase in the mean ages. The age ranges in the present study and the study by Olze et al. [19] are almost identical. The mean ages of mineralization stages D to H are also very similar. The age interval in the study by Cavrić et al. [20] is lower than that in the present study. The mean ages are also consistently lower. The sample from Uys et al. [17] has a higher lower age limit and a lower upper age limit than the sample in the present study. Consequently, the mean ages in the lower stages are higher and in stage H lower than in the present study.

If exceeding legally relevant age limits must be proven with the highest standard of evidence, the so-called minimum age concept is applied. The minimum age is derived from the minimum age in the reference study used for the identified characteristic. This is therefore the age of the youngest person in the reference population who exhibits the respective stage of development [2]. Since many studies are not based on a sufficient number of cases, several valid studies must be taken into account for each characteristic. From these studies, the one with the lowest minimum age must then be selected.

For the most forensically significant age limit of 18 years, the minimum age of stage H is relevant. In this study, the minimum age in stage H is just above 18 years for tooth 48, while it is just below 17.9 years for tooth 38. For females, only the minimum age for tooth 38 in stage H is above 18 years. This value is rounded to 18.1 years. For tooth 48, the value is 17.2 years, which is below 18 years. These minimum ages are above the values reported in other studies and therefore cannot be recommended for age assessment practice. In the study by Cavrić et al. [20], the minimum ages are surprisingly low at 15.7 years for males and 15.1 years for females. In addition to different case numbers, incorrect stage determinations may also be a reason for the differing minimum ages. These are particularly problematic when applying the minimum age principle. In most studies to date, all stage determinations were made by a single examiner. To determine intra-observer agreement, some or all of the cases were re-evaluated by the same examiner. To determine inter-observer agreement, some or all of the cases were evaluated by a second examiner. The agreement was never 100%, which means that there must have been misclassifications. The first determinations of the first examiner were generally used to calculate the statistical measures. It can be assumed that this approach resulted in misclassifications being included in the evaluations. To minimize such misclassifications, stage determination in our study was performed by consensus.

## Conclusions

This study presents reference values for dental age determination based on third molar mineralization in individuals from sub-Saharan Africa with confirmed dates of birth. The minimum ages determined for mineralization stage H are above the values of previous studies and therefore cannot be recommended for age assessment practice. In order to avoid stage misclassifications, which are particularly problematic when applying the minimum age principle, experienced examiners should make stage classifications in future studies consensually by.
